# How severe and prevalent are Ebola and Marburg viruses? A systematic review and meta-analysis of the case fatality rates and seroprevalence

**DOI:** 10.1186/s12879-016-2045-6

**Published:** 2016-11-25

**Authors:** Luke Nyakarahuka, Clovice Kankya, Randi Krontveit, Benjamin Mayer, Frank N. Mwiine, Julius Lutwama, Eystein Skjerve

**Affiliations:** 1 Norwegian University of Life Sciences, Oslo, Norway; 2 Makerere University, Kampala, Uganda; 3 Norwegian Medicines Agency, Oslo, Norway; 4 Ulm University, Ulm, Germany; 5 Uganda Virus Research Institute, Entebbe, Uganda

**Keywords:** Ebola virus disease, Marburg virus disease, Case fatality rate, Meta-analysis, Systematic review, Seroprevalence

## Abstract

**Background:**

Ebola and Marburg virus diseases are said to occur at a low prevalence, but are very severe diseases with high lethalities. The fatality rates reported in different outbreaks ranged from 24–100%. In addition, sero-surveys conducted have shown different seropositivity for both Ebola and Marburg viruses. We aimed to use a meta-analysis approach to estimate the case fatality and seroprevalence rates of these filoviruses, providing vital information for epidemic response and preparedness in countries affected by these diseases.

**Methods:**

Published literature was retrieved through a search of databases. Articles were included if they reported number of deaths, cases, and seropositivity. We further cross-referenced with ministries of health, WHO and CDC databases. The effect size was proportion represented by case fatality rate (CFR) and seroprevalence. Analysis was done using the *metaprop* command in STATA.

**Results:**

The weighted average CFR of Ebola virus disease was estimated to be 65.0% [95% CI (54.0–76.0%), I^2^ = 97.98%] whereas that of Marburg virus disease was 53.8% (26.5–80.0%, I^2^ = 88.6%). The overall seroprevalence of Ebola virus was 8.0% (5.0%–11.0%, I^2^ = 98.7%), whereas that for Marburg virus was 1.2% (0.5–2.0%, I^2^ = 94.8%). The most severe species of ebolavirus was *Zaire ebolavirus* while *Bundibugyo Ebolavirus* was the least severe.

**Conclusions:**

The pooled CFR and seroprevalence for Ebola and Marburg viruses were found to be lower than usually reported, with species differences despite high heterogeneity between studies. Countries with an improved health surveillance and epidemic response have lower CFR, thereby indicating need for improving early detection and epidemic response in filovirus outbreaks.

## Background

Ebola virus disease (EVD) and Marburg virus disease (MVD) are caused by filoviruses in the family *Filoviridae* and are both associated with high case fatality rates (CFR). The World Health organization (WHO) reports that the CFR of EVD ranges from 25.0 to 90.0% while that of MVD ranges from 24.0 to 88.0% [1]. In the early phases of a major Ebola outbreak in West Africa, CFR was reported to be 70.8% [2]. The CFR of EVD seems to be species dependent with *Ebola Zaire* and *Ebola Sudan* species being most pathogenic (with a reported CFR of 100%), while *Ebola Bundibugyo* appears to have a lower CFR at 34% [3]. A recent study by Lefebvre *et al.* that used data from WHO database estimated the CFR of EVD to be 65.4% irrespective of the Ebola virus species [4]. A few studies have tried to pool the CFR of EVD and MVD, but did not use the meta-analysis approach [5].

Although EVD is known to be very severe, there are some species of Ebola virus that cause less serious disease. For example, *Taï Forest ebolavirus,* formerly known as *Côte d’Ivoire ebolavirus*, has not been associated with any fatality and the only case ever reported recovered from the disease [6]. While there have been some reports of EVD being associated with a CFR of 100%, this CFR is attributed to only a single case fatality that did not result into transmission of the virus to other individuals [7, 8]. It seems that CFR differs from species to species, however, both *Ebola Sudan* and *Ebola Zaire* have shown a CFR of 100% [1]. Also, the CFR of the MVD outbreak that occurred in Uganda in 2014 was reported to be 100%, but again only one person was diagnosed and died from the disease [9]. The largest MVD outbreak was in Angola in 2004 with CFR of 90% [10] and in Democratic Republic of Congo (DRC) in 1998 with CFR of 83% [11].

There is evidence that a substantial proportion of infected humans in Central Africa seem to recover without being detected by the health care system, and apparently healthy individuals have been found to be seropositive for Ebola and Marburg viruses [12–15]. Furthermore, Marburg virus has been found in apparently healthy cave-dwelling fruit bats of species *rousettus aegyptiacus,* which are believed to be reservoirs for Marburg virus*,* and responsible for the spill over into human populations [16–19]. Because of the variations in the reported CFR and the presence of seropositive individuals, it is important to determine the severity and prevalence of these viral haemorrhagic fevers. This is important for forecasts and risk analysis especially during outbreaks for epidemic preparedness and response by affected countries. This will help to estimate how many infected people with EVD or MVD are likely to die from the disease during outbreaks. Whereas there are few studies that have estimated CFR of EVD [4, 5], these did not use a meta-analysis approach and no meta-analysis has been performed on CFR of EVD, MVD, seroprevalence of Ebola and Marburg viruses. Therefore, our aim was to determine the overall weighted estimate (effect size) of the CFR and seroprevalence of EVD and MVD using available published literature on outbreak reports, WHO and CDC databases and population based studies for seroprevalence of filoviruses (Marburg and Ebola viruses). We also explored whether CFR and seroprevalence of these filoviruses differs according to virus species and country.

## Methods

Procedures for systematic reviews and meta-analysis have been developed to summarize scientific evidence from the literature. This work was done following the guidelines published in the PRISMA statement [20] and MOOSE guidelines for observational studies [21] as follows.

### Literature search strategy

A detailed literature search was conducted by the authors in PubMed (as well as Medline), Web of Science and Google Scholar until 5^th^ October 2015. In cases where there was no peer-reviewed publication for a known outbreak, data was retrieved from websites of WHO and CDC. The following key words were used; “ebola”, “ebolavirus”, “viral haemorrhagic fevers”, “marburg virus disease”, “marburg haemorrhagic fever”, “marburg virus outbreak”, “ebola virus disease outbreak”, “marburg virus”, “ebola outbreak”, “seroprevalence of ebola virus”, “seroprevalence of marburg virus” and “risk factors of viral haemorrhagic fevers”. The search included all articles and outbreak reports about EVD and MVD and cross-referencing of primary articles was done to obtain the original articles. Since the number of outbreaks of EVD and MVD are known and few, efforts were made to obtain all information about these outbreaks from WHO and CDC websites and Ministries of health of respective countries.

### Study selection criteria

**Fig. 1:**
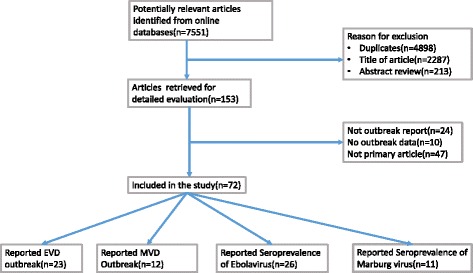
Flow diagram for search strategy and article selection process from the literature databases

Studies were included in the meta-analysis if they reported the total number of cases and total number of deaths from the outbreak of EVD or MVD. Also studies that were reporting CFR and sero-prevalence in percentages were included. Studies or reports that did not include total number of deaths or cases were excluded as well as studies that did not report original data (Fig. 1). We also excluded studies that reported outbreaks of Ebola species that are not pathogenic to humans and those species that have not caused mortality in humans. In cases where there were multiple publications, we used the one with the most complete data or the most recent one. In cases where there was controversy on the number of cases and deaths between studies, we cross-referenced with the respective ministries of health, WHO or CDC databases to reconcile these discrepancies. Seroprevalence studies included were only those that were population based and comprised apparently healthy individuals. We excluded articles that reported sero-prevalence during outbreaks or in sick individuals.

### Data extraction

LN compiled a list of articles and discrepancies were discussed and resolved by consensus between FM, CK and JL. We used a standardized data extraction form and the following information was extracted for each qualifying study and outbreak report: i) author; ii) Country; iii) number of cases; iv) number of deaths; v) CFR (if reported); vi) month and year of outbreak; vii) year of publication viii) and species involved. For population-based sero-prevalence studies, the following additional information was retrieved: i) sample size and ii) number of seropositive samples.

### Statistical analysis

Data were collected in a Microsoft Excel® spreadsheet and outcome measures were calculated. CFR was calculated as number of deaths divided by reported cases whereas seroprevalence was calculated as number of individuals seropositive divided by total sample size in each study. Our effect size (ES), the principal summary measure, was the proportion represented by CFR and seroprevalence. We used the newly developed *metaprop* command [22] for performing meta-analysis of binomial data in STATA (StataCorp, College Station, TX, USA). The *metaprop* command was preferred to *metan* command because it implements procedures that are specific to binomial data and is appropriate for dealing with proportions close to or at the margins and also uses the Freeman-Tukey double arcsine transformations to stabilize the variances [22]. The meta-analysis of CFR was stratified by country and species where possible.

The following parameters were estimated: Cochran’s Q indicating differences in true ESs, an estimate of the true variance of ESs between studies (our estimate of τ^2^) and Higgins I^2^ which is an estimate of what proportion of the observed variance that reflects real differences in ES. If I^2^ is close to 0, then almost all the observed variation is spurious, and there is nothing to explain. If I^2^ is large, then reasons for the observed variance should be evaluated [23, 24]. Sensitivity analysis was done by excluding studies that reported very few numbers or zero deaths or no seropositives. A meta-regression procedure was done to assess if factors such as species, country, year and month of outbreak influence CFR of both EVD and MVD using the traditional logit-transformation: Logit (prevalence) = ln [prevalence/ (1 − prevalence)] Variance (logit) =1/ (np) +1/[n (1 − p)] [25]. The Begg’s and Egger’s tests were used in combination with a funnel plot to assess potential publication bias and visualised using funnel plots [24, 26].

## Results

### Literature search result

Results from the literature search are illustrated in Fig. 1. The literature search yielded 7551 articles. Of these, 4898 were excluded as duplicates. After reviewing the titles and the abstract, only 153 articles were retrieved for detailed evaluation. After full evaluation of retrieved publications, 72 articles were included in this study. Of those included in the study, 23 reported outbreaks of EVD (Table 1) [3, 8, 27–41, 7, 42, 43], 12 reported outbreaks of MVD (Table 2) [10, 11, 42, 44–51], 26 reported sero-prevalence of Ebola virus (Table 3) [8, 12–14, 28, 31, 52–54, 29, 55–70] and 11 reported sero-prevalence of Marburg virus (Table 4) [14, 15, 57, 61–64, 67, 71–73]. Most of the sero-prevalence studies reported both Marburg and Ebola viruses.

**Table 1:** Summary of the studies included in a systematic review and meta-analysis describing case fatality rate for Ebola virus disease in Africa

Author and Year of Publication	Deaths	Cases	Country	Year and month of outbreak
WHO International Study Team, 1978 [27]	151	284	South Sudan	1976, June–November
International Commission, 1978 [28]	280	318	DRC	1976, Sept–Oct
Heymann et al., 1980 [8]	1	1	DRC	1977, June
Baron et al., 1983 [29]	22	34	South Sudan	1979, June–Oct
Amblard et al., 1997 [30]	30	49	Gabon	1994, November
Khan et al., 1999 [32]	255	315	DRC	1995, May
Georges et al., 1999 [31]	21	31	Gabon	1996, May
Milleliri et al., 2004 [34]	45	60	Gabon	1996, May
Okware et al., 2002 [33]	224	425	Uganda	2000, October
Nkoghe et al., 2005 [36]	97	124	Gabon	2000, December
Rouquet et al. (2005) [37]	128	143	ROC	2003, December
Boumandouki et al., 2005 [35]	29	35	ROC	2003, Oct–Dec
Onyango et al., 2007 [38]	7	17	South Sudan	2004, April–June
Nkoghe et al., 2011 [41]	10	12	ROC	2005, April–May
Leroy et al., 2009 [39]	186	264	DRC	2007, May and November
Wamala et al., 2010 [3]	39	116	Uganda	2007, August
Grard et al., 2011 [40]	15	32	DRC	2008, Jan
Shoemaker et al., 2012 [7]	1	1	Uganda	2011, May
Albariño et al., 2013 [42]	4	11	Uganda	2012, July
Albariño et al., 2013 [42]	3	6	Uganda	2012, Nov
Albariño et al., 2013 [42]	13	36	DRC	2012, August
Maganga et al., 2014 [43]	49	69	DRC	2014, July
WHO, 2016 [79, 90]	11323	28646	West Africa	March, 2014

*DRC* Democratic Republic of Congo, *ROC* Republic of Congo

**Table 2:** Summary of studies included in a systematic review and meta-analysis describing case fatality rate for Marburg virus from searched literature globally

Author and Year of Publication	Deaths	Cases	Country	Year & Month of outbreak
Siegert, 1972 [44, 45]	7	31	Germany and Yugoslavia	1967, August
Gear et al., 1975 [91]	1	3	Johannesburg, South Africa	1975, February
Smith et al., 1982 [92]	1	2	Kenya	1980, January
Johnson et al., 1996 [49]	1	1	Kenya	1987, August
Nikiforov et al., 1994 [48]	1	1	Russia	1990
Bausch et al., 2006 [11]	128	154	DRC	1998, October
Towner et al., 2006 [10]	227	252	Angola	2004, October
Adjemian et al., 2011 [51]	1	4	Uganda	2007, June
Centers for Disease & Prevention, 2009 [50]	0	1	USA from Uganda	2008, January
Timen et al., 2009 [93]	1	1	Netherlands from Uganda	2008, July
Albarino et al., 2013 [42, 94]	4	15	Uganda	2012, October
WHO, 2015 [95]	1	1	Uganda	2014, October

*DRC* Democratic Republic of Congo

**Table 3:** Summary of studies included in a systematic review and meta-analysis describing sero-prevalence of Ebola virus from literature

Author and Year of Publication	Sample size	Seropositive	Country
Van der Groen and Pattyn 1979 [96]	251	43	DRC
Saluzzo, Gonzalez et al. 1980 [97]	499	17	CAR
Bouree & Bergmann, 1983 [55]	1517	147	Cameroon
Johnson et al., 1983 [56]	741	8	Kenya
Van der Waals, Pomeroy et al. 1986 [57]	225	30	Liberia
Meunier et al., 1987 [58]	1528	319	CAR
Paix et al., 1988 [59]	375	4	Cameroon
Tomori, Fabiyi et al. 1988 [60]	1,677	30	Nigeria
Gonzalez et al., 1989 [72]	5070	629	Central Africa
Mathiot, Fontenille et al. 1989 [61]	381	17	Madagascar
Johnson, Gonzalez et al.1993a [63]	427	75	CAR
Johnson, Gonzalez et al. 1993b [64]	4295	914	CAR
Busico et al., 1999 [66]	575	24	DRC
Nakounne, Selekon et al. 2000 [67]	1762	104	CAR
Heffernan et al., 2005 [69]	979	14	Gabon
Allela et al., 2005 [68]	439	64	Gabon
Lahm, Kombila et al. 2007 [70]	1147	14	Gabon
Becquart et al., 2010 [12]	4349	665	DRC
Heymann et al., 1980 [8]	1096	79	DRC
Burke et al., 1978 [28]	984	38	DRC
Baron et al., 1983 [29]	106	23	Sudan
Georges et al., 1999 [31]	441	58	Gabon
Becker, Feldmann et al. 1992 [62]	1288	11	Germany
Gonzalez, Nakoune et al. 2000 [14]	1331	71	CAR
Bertherat, Renaut et al. 1999 [65]	236	24	Gabon
Nkoghe, Padilla et al. 2011 [13]	4349	667	DRC

*DRC* Democratic Republic of Congo, *ROC* Republic of Congo, *CAR* Central African Republic

**Table 4:** Summary of studies included in a systematic review and meta-analysis describing sero-prevalence of Marburg disease from published literature

Author and Year of Publication	Sample size	Seropositive	Country
Van der Waals, Pomeroy et al. 1986 [57]	225	3	Liberia
Gonzalez, Josse et al. 1989 [72]	5070	20	Central African countries
Johnson, Ocheng et al. 1983 [71]	1899	8	Kenya
Mathiot, Fontenille et al. 1989) [61]	384	0	Madagascar
Becker, Feldmann et al. 1992 [62]	1288	34	Germany
Johnson, Gonzalez et al. 199a [63]	427	5	CAR
Johnson, Gonzalez et al. 1993b [64]	4295	137	CAR
Gonzalez, Nakoune et al. 2000 [14]	1340	33	CAR
Nakounne, Selekon et al. 2000 [67]	1762	35	CAR
Bausch, Borchert et al. 2003 [15]	912	15	DRC
Borchert, Mulangu et al. 2006 [73]	300	0	DRC

*DRC* Democratic Republic of Congo, *CAR* Central African Republic

Two more outbreaks have occurred without human mortalities namely Ebola Reston [74, 75] and another caused by *Taï* Forest virus [6]. *Zaire ebolavirus* species was responsible for most of the outbreaks with 14/23 (60.9%) [8, 28, 30–32, 34–36, 39, 40, 41, 37, 76] followed by *Sudan ebolavirus* with 30.3% (7/23) outbreaks [27, 29, 38, 7, 42, 77] and lastly *Bundibugyo ebolavirus* 8.7% (2/23) [3, 42]. Most articles reported DRC (7/23) [8, 28, 32, 39, 40, 42, 76] and Uganda (5/23) [3, 33, 7, 42] as countries most affected by EVD outbreaks. Other countries reported include Gabon (4/23) [31, 34, 36, 78], Republic of Congo (3/23) [35, 37, 41], South Sudan (3/23) [27, 29, 38] and multiple countries in West Africa associated with the recent single outbreak [79–82]. Interestingly, most of the EVD outbreaks occurred during months of May, June and July and no outbreaks were reported in the month of February.

### Meta-analysis and meta-regression of CFR and seroprevalence of EVD

**Fig. 2:**
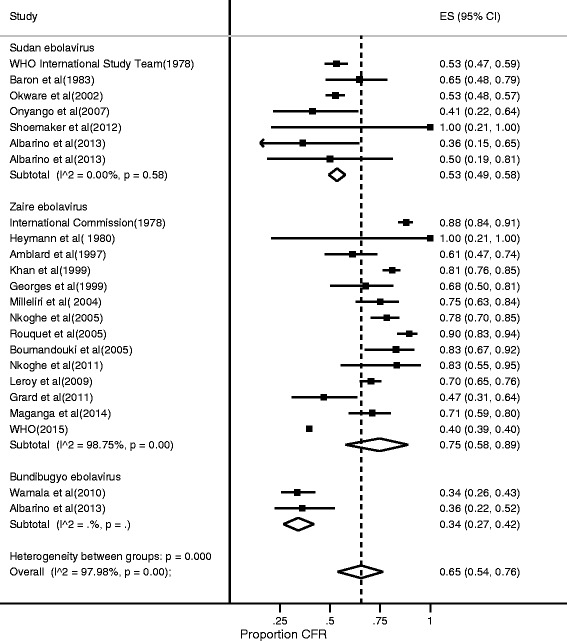
Forest plot showing stratified meta-analysis of CFR of Ebola Virus Disease by virus species estimated by the random effects model (I^2^ = Higgins statistic, ES = Effect size, CI = Confidence Interval)

**Fig. 3:**
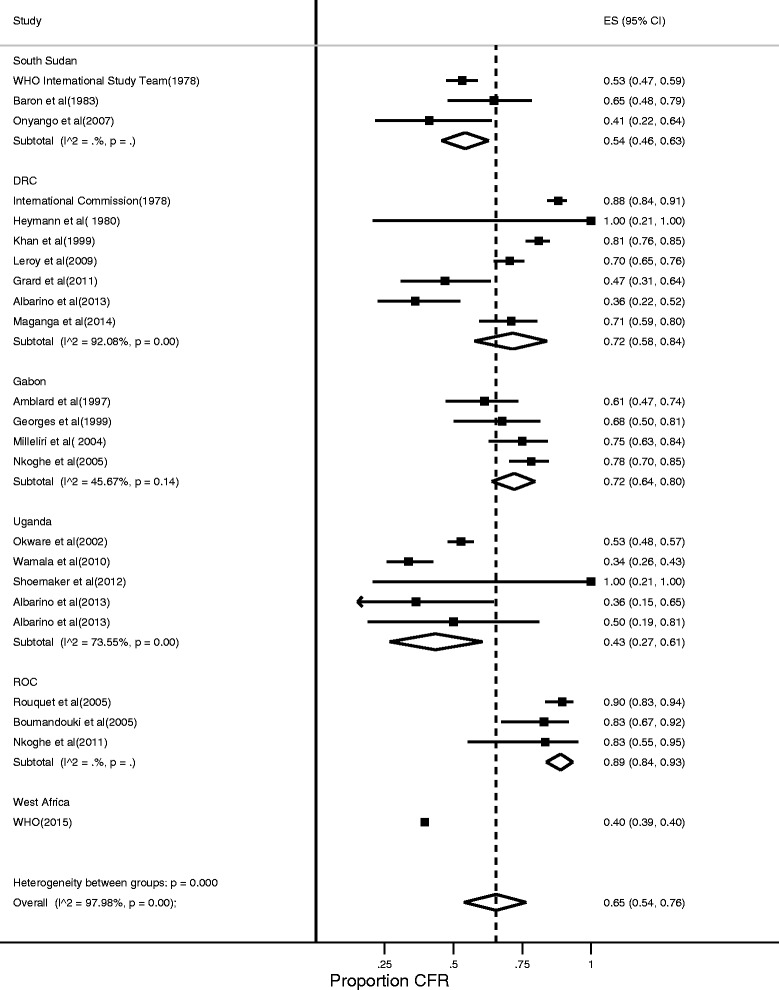
Forest plot showing stratified meta-analysis of CFR of Ebola virus disease by country estimated by the random effects model (I^2^ = Higgins statistic, ES = Effect size, CI = Confidence Interval, DRC = Democratic Republic of Congo, ROC = Republic of Congo)

**Fig. 4:**
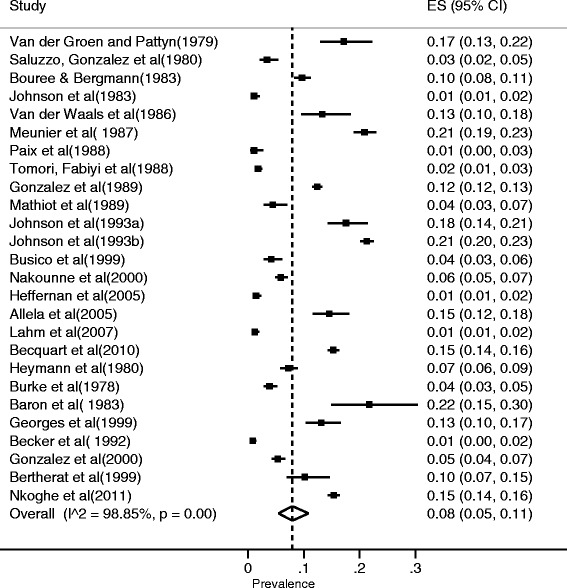
Forest plot for the meta-analysis of sero-prevalence studies of Ebola virus (I^2^ = Higgins statistic, ES = Effect size, CI = Confidence Interval)

The weighted CFR of EVD from 23 outbreaks was 65% (95% CI: 54–76%) (Fig. 2). There was a substantial between-study variance indicating heterogeneity in the overall CFR of EVD, I^2^ = 97.98%. On stratification by Ebola virus species, the CFR for *Sudan ebolavirus* was 53%, *Bundibugyo ebolavirus* was 34%, whereas that of *Zaire ebolavirus* was 75%. From the meta-regression, the CFR for *Zaire ebolavirus* was higher compared to other Ebola species (=0.006, Coefficient = 0.19, 95% CI = 0.063 - 0.588). In sub-analysis analysis by country, the highest CFR for EVD was observed in Republic of Congo (89.0%, 84.0–93.0%) whereas the lowest was found in Uganda (43.0%, 27.0–61.0%) (Fig. 3). However, the large West African EVD outbreak that affected multiple countries had an even lower CFR at 40% (39–40%). The pooled ES for Ebola virus seroprevalence was 8% [5–11%) with substantial between-study variance (I^2^ = 98.7%) (Fig. 4).

### Meta-analysis and meta-regression of CFR and seroprevalence of MVD

**Fig. 5:**
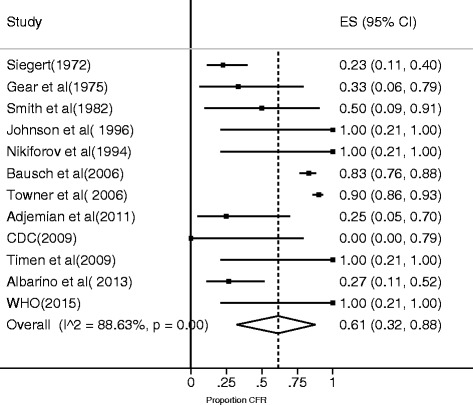
Forest plot for a meta-analysis of CFR of Marburg virus disease estimated using a random effects model (I^2^ = Higgins statistic, ES = Effect size, CI = Confidence Interval)

**Fig. 6:**
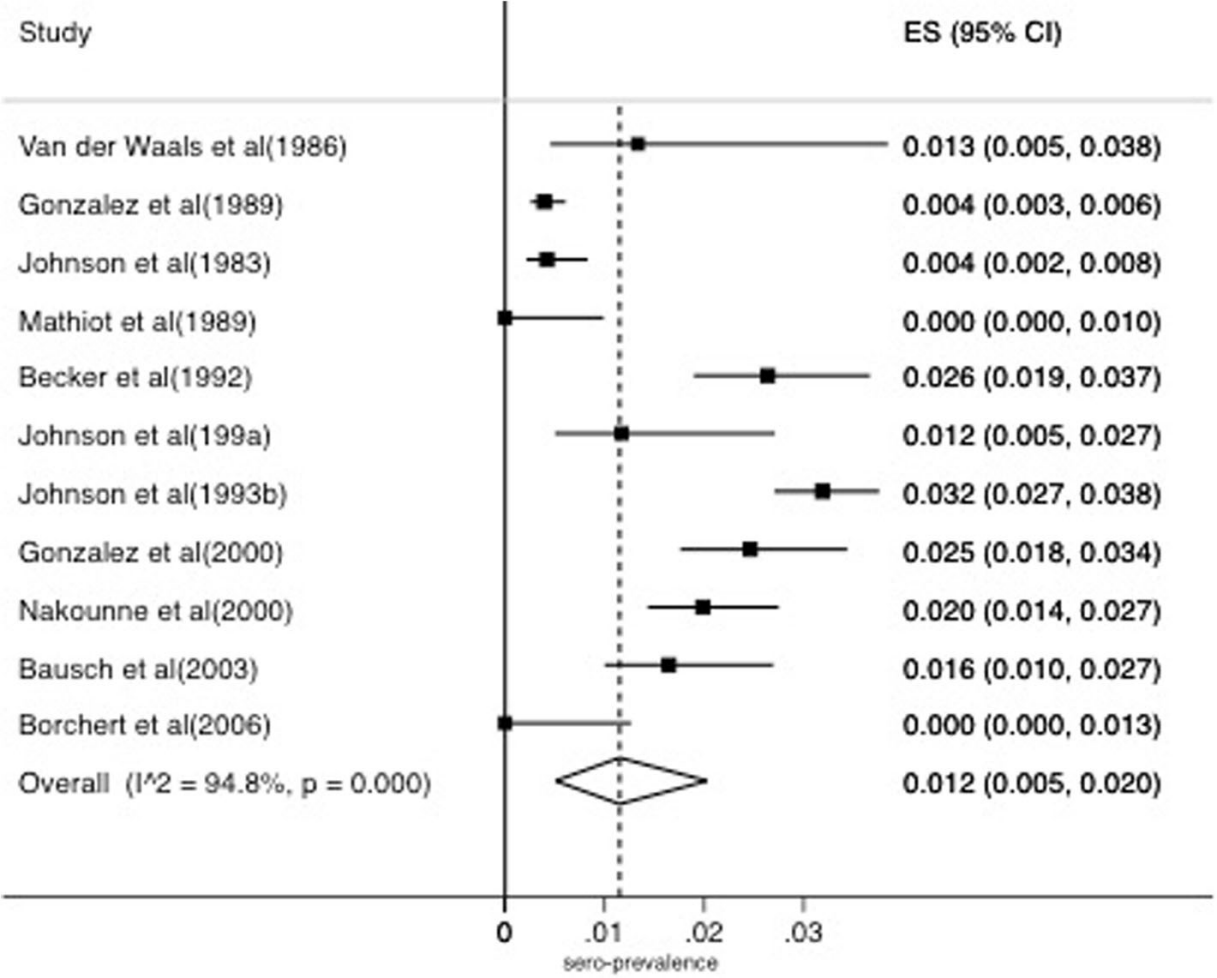
Meta-analysis of seroprevalence of Marburg virus estimated using a random effects model (I^2^ = Higgins statistic, ES = Effect size, CI = Confidence Interval)

The MVD CFR was lower than that of EVD (61%) (Fig. 5). There was no significant difference between CFR of MVD and different variables in the meta-regression model (*P* = 0.637). The pooled seroprevalence of Marburg virus was lower than that of Ebola virus at 1.2% (0.5–2%) (Fig. 6).

### Publication bias

**Fig. 7:**
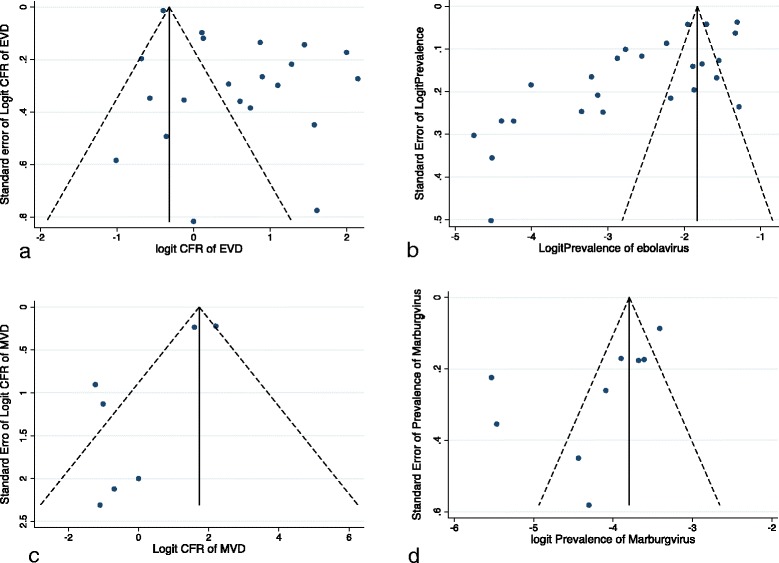
Funnel plots assessing publication bias in studies reporting case fatality rate and seroprevalence of Ebola virus disease and Marburg virus disease. **a** Funnel plot of the point estimates of the logit CFR of EVD, **b** Funnel plot of the point estimates of the logit prevalence of EVD, **c** Funnel plot of the point estimates of the logit CFR of MVD, **d** Funnel plot of the point estimates of the logit prevalence of MVD

In the funnel plots, asymmetry was evident which gives rise to suspected publication bias (Fig. 7). Egger’s test was significant for studies reporting CFR and seroprevalence of EVD and MVD (*P* = 0.001, *P* < 0.001, *p* = 0.032, and 0.046 respectively). However, the Begg’s bias test was not significant for studies reporting CFR of EVD and MVD (*p* = 0.091 and *p* = 0.293 respectively), seroprevalence of MVD (*p* = 0.95), but was significant for studies reporting seroprevalence of EVD (*p* = 0.007).

## Discussion

Our findings show that the overall pooled CFR of EVD of 65% was lower than the previously reported CFR of 90% [83]. This indicates, despite substantial heterogeneity, that more than half of the individuals who contract EVD are more likely to die. Although this CFR appears to be high, it is lower than the exaggerated figure of 90%. This high CFR tends to cause fear and panic in the general public and hence interferes with response mechanisms [84]. The CFR in our study is similar to that reported by Lefebvre *et al.* [4], who reported a CFR of 65% in a study done using WHO database on EVD outbreaks. Although there have been cases of EVD and MVD with 100% CFR [8, 7], these were isolated single cases that should not be generalized by scientific community to consider Ebola and Marburg viruses as highly virulent diseases with CFR of up to 90%. There have been reports with a higher CFR than our maximum of 76% [28, 35, 37, 41], but these either happened long time ago [28] where there was little knowledge about the disease or happened in very remote places where health care delivery systems are not robust.

The high CFR of EVD in Republic of Congo (89%) compared to Uganda (43%) may be due to partly, differences in health care system and response mechanisms to outbreaks, but also the severity of the species of Ebola virus involved. For example, Uganda has developed a robust surveillance system for detecting these viral haemorrhagic fevers and epidemic response is started within hours of a positive diagnosis at a CDC supported laboratory in the country [85]. The well-established disease surveillance system and organised health care delivery in endemic areas might explain the lower CFR for EVD observed in Uganda. But it is also important to note that Uganda has been affected by the less pathogenic species of Ebola virus (*Sudan ebolavirus* and *Bundibugyo ebolavirus*) as compared to DRC and West African countries that have experienced *Zaire ebolavirus* Also, it is important to look at the denominators and numerators when interpreting the CFR. In this analysis, we see that CFR of EVD in a large outbreak in West Africa that affected multiple countries is at CFR of 40% using WHO data, but this alone would be misleading if the real numbers of deaths and cases were not looked at. As of 30^th^ March 2016, there were 11323 deaths and 28646 cases due to EVD from all countries affected by that outbreak.

Another significant finding of our study was the variation in the severity and CFR among the pathogenic species of Ebola virus. Zaire *ebolavirus* (CFR, 75%) was found to be the most severe followed by *Sudan ebolavirus* (CFR, 53%), while *Bundibugyo ebolavirius* (CFR, 34%) was the least severe species. This finding is supported by McCormick *et al.*, who described differences in severity and filovirus dynamics [86, 87]. The reasons for severity of *Zaire ebolavirus* are unclear, thus there is a need for further research to determine whether genetic differences are responsible for the variation in pathogenesis of these species. There was also heterogeneity within *Zaire ebolavirus* outbreaks (P < 0.001) meaning that these outbreaks, although caused by the same species are not always similar. The heterogeneity could further be explained by differences in outbreak investigation designs or approaches, location of the outbreak and data collection methods. This is further supported by the strains that have been found within Ebola Zaire species [40]. There was less heterogeneity in outbreak reports for *Bundibugyo ebolavirus* and *Sudan ebolavirus* probably due to few outbreaks that have been caused by these species. However, the meta-regression did not show any influence on CFR of EVD by country of outbreak (*p* = 0.249). This is probably due to low power given the few number of outbreaks that we have had globally.

With the *Metaprop* command for meta-analysis of marginal proportions [22], it was possible to estimate the 95% confidence intervals for MVD as 61% (32–88%). The CI was very wide because of the few outbreaks and the number of cases involved in MVD outbreaks as compared to EVD outbreaks. Dropping studies with 100% or 0% CFR for MVD, the CFR reduced from 61 to 53%. With few outbreaks of Marburg virus in different countries, there is a high variation that would impact the estimation of CFR for MVD, but this was not significant from the meta-regression (*p* = 0.913).

We found that apparently healthy individuals in central African countries, that are endemic for viral haemorrhagic fevers, had a 5 and 1% chance of having antibodies against Ebola and Marburg viruses, respectively. This finding suggests that some individuals who get infected with filoviruses make a full recovery without severe complications and being documented by healthcare systems. Although the sero-prevalence is low, it is important that these seropositive individuals are detected early enough because of greater mortality and socio-economic implications associated with these infections. Because serological tests have been reported to have low specificity and there is a lot of cross-reactivity of filoviruses with other viral haemorrhagic fevers [88], this finding should be interpreted with caution. It is important that specific and more accurate tests are developed to accurately measure antibody response against filoviruses and progress in this direction has been made due to the recently approved rapid diagnostic test for Ebola virus by WHO [89].

The limitation of our ES estimates was the heterogeneity that was observed between studies. Efforts to identify sources of heterogeneity were made, and many unmeasured factors could have influenced CFR during outbreaks. These reports had data that were collected using different methods and hence combining them to produce one effect was likely to produce high heterogeneity. Sensitivity analysis by dropping single cases with 100% mortality did not have substantial impact on the result. Funnel plots and Beggs tests suggested that publication bias might have been present, meaning that studies with negative results about Ebola and Marburg viruses are less likely to be published hence affecting the estimate of seroprevalence and CFR for EVD and MVD.

The fact that laboratory tests for Ebola and Marburg viruses are expensive, used only in specific laboratories and that serological tests are not specific might influence the publication of studies done with these tests.

## Conclusions

The CFR for Ebola and Marburg viruses is still moderately high but not as high as has been reported in the media and other publications. The CFR of EVD and MVD is higher in countries with poor disease surveillance systems. This calls for an improved surveillance system that will enhance early detection and response to these filovirus outbreaks to avoid a pandemic. The presence of seropositive individuals in apparently health populations indicate that cases go undetected by the health care system in affected countries; further calling for robust surveillance for Ebola and Marburg viruses.

## Abbreviations

CDC: Centres for disease control and prevention USA
CFR: Case fatality rate
CI: Confidence interval
DRC: Democratic Republic of Congo
EVD: Ebola virus disease
MVD: Marburg virus disease
ROC: Republic of Congo
WHO: World Health Organization
